# Characterization of In Vitro and In Vivo Metabolism of Antazoline Using Liquid Chromatography-Tandem Mass Spectrometry

**DOI:** 10.3390/ijms21249693

**Published:** 2020-12-18

**Authors:** Joanna Giebułtowicz, Natalia Korytowska, Roman Piotrowski, Piotr Kułakowski, Gniewomir Latacz, Ewa Szymańska, Barbara Wiśniowska, Sebastian Polak

**Affiliations:** 1 Department of Bioanalysis and Drugs Analysis, Faculty of Pharmacy, Medical University of Warsaw, Banacha 1, 02-097 Warsaw, Poland; natalia.korytowska@wum.edu.pl; 2 Department of Cardiology, Postgraduate Medical School, Grochowski Hospital, 04-073 Warsaw, Poland; rpiotrow@op.pl (R.P.); kulak@kkcmkp.pl (P.K.); 3 Department of Technology and Biotechnology of Drugs, Faculty of Pharmacy, Jagiellonian University Medical College, Medyczna 9, 30-688 Kraków, Poland; gniewomir.latacz@uj.edu.pl (G.L.); ewa.szymanska@uj.edu.pl (E.S.); 4 Department of Social Pharmacy, Faculty of Pharmacy, Jagiellonian University Medical College, Medyczna 9, 30-688 Kraków, Poland; b.wisniowska@uj.edu.pl (B.W.); sebastian.polak@uj.edu.pl (S.P.); 5 Certara UK Limited, Simcyp Division, Sheffield S1 2BJ, UK

**Keywords:** antazoline, atrial fibrillation, metabolism, cytochrome P450 isoform, liquid chromatography coupled with tandem mass spectrometry

## Abstract

Antazoline (ANT) was recently shown to be an effective and safe antiarrhythmic drug in the termination of atrial fibrillation. However, the drug is still not listed in clinical guidelines. No data on ANT metabolism in humans is available. We used liquid chromatography coupled with tandem mass spectrometry to identify and characterize metabolites of ANT. We analyzed plasma of volunteers following a single intravenous administration of 100 mg of ANT mesylate and in in vitro cultures of human hepatocytes. We revealed that ANT was transformed into at least 15 metabolites and we investigated the role of cytochrome P450 isoforms. CYP2D6 was the main one involved in the fast metabolism of ANT. The biotransformation of ANT by CYP2C19 was much slower. The main Phase I metabolite was M1 formed by the removal of phenyl and metabolite M2 with hydroxyl in the *para* position of phenyl. Glucuronidation was the leading Phase II metabolism. Further study on pharmacokinetics of the metabolites would allow us to better understand the activity profile of ANT and to predict its potential clinical applications. Ultimately, further investigation of the activity profile of the new hydroxylated M2 metabolite of ANT might result in an active substance with a different pharmacological profile than the parent molecule, and potentially a new drug candidate.

## 1. Introduction

Antazoline (ANT) is a first-generation antiarrhythmic and anticholinergic agent that is well-established in clinics. It was originally used for the management of acute allergic reactions [1]. However, recent data showed that ANT is also very effective in the termination of atrial fibrillation (AF) [2,3,4]. Recently, retrospective analysis of the randomized controlled trial has been conducted and showed high effectiveness of ANT in the rapid conversion of recent-onset AF to sinus rhythm [2]. Additionally, ANT was effective in the termination of AF during accessory pathway ablation [5] as well as pulmonary veins isolation [6]. ANT was also shown as an effective and safe option in the termination of AF in patients with stable coronary artery disease [7]. ANT is usually well tolerated, however, some mild side effects occurred. The most common side effects of ANT include transient heat sensation in the throat and lower abdomen, vertigo, hypotension, or nausea, and vomiting [6,8]. There has been a case report on ANT causing granulocytopenia. However, in addition to ANT, the patient was given penicillamine, which might cause granulocytopenia, or it could be due to the additive effect of penicillamine and ANT [9].

Despite numerous data showing that ANT is an effective and safe antiarrhythmic drug, it is still not listed in the clinical guidelines [10]. Most of the information about ANT is based on clinical data. During the last decade, new data emerged and shed more light on the antiarrhythmic function of ANT. The first randomized study was conducted which confirmed the high efficacy and safety of ANT in the termination of AF [2]. Recently, data on the pharmacokinetics of single intravenous administration of 100 mg of ANT mesylate in healthy volunteers presented the fast elimination and the relatively high volume of distribution of the drug [11]. ANT also prolonged P wave, QRS duration, and QT/QTc, which corresponds with drug-induced prolongation of conduction (P wave and QRS) and repolarization (QT/QTc) [8]. During the ablation of supraventricular arrhythmias, ANT led to an increase in sinus rhythm and prolonged interatrial conduction [12]. A lack of impact of ANT on conduction between pulmonary vein potentials and atrial muscle was also shown [13]. Moreover, in an animal model of the drug-induced increased atrial refractory period, ANT reduced interatrial conduction time which was associated with high AF suppression rate [14]. However, many questions regarding the mechanism of action of ANT remain to be answered. For instance, it is not known whether ANT is the active agent itself, or the pharmacological effects are also related to some active metabolites. Scarce data on the metabolic profile of ANT and no data on their activities are available.

Metabolism is the process of biotransformation of a drug substance by various bodily systems. Two phases of the metabolism of pharmaceuticals are frequently recognized. Phase I is carried out mostly by microsomal monooxygenases, with the main role played by cytochrome P450 (CYP P450). This phase predominantly involves hydrolysis, reduction, and oxidation, and it introduces a polar substituent into the molecule. Phase II includes glucuronide, sulfate, glutathione, and amino acid conjugation as well as acetylation and methylation to form water-soluble products, usually less toxic and more easily excreted than the parent drug substance [15]. It is essential to evaluate the pharmacodynamic consequences of these metabolic reactions. Biotransformation mainly involves inactivation of the xenobiotics. It can also result in the formation of the active metabolite (e.g., in case of leflunomide). Moreover, due to higher hydrophilicity, the metabolite can demonstrate better safety profile than the parent drug (as observed for terfenadine and its metabolite—fexofenadine). Regulatory authorities recommend the study on drug metabolites [16,17] in the safety assessment of the drug, thus the determination and characterization of systemic metabolites are required. The metabolism of ANT was investigated using only rabbit liver microsomal fraction [18]. *N*-benzylaniline and *p*-benzylaminophenol were detected as metabolic products [18]. No data on ANT metabolism in humans is available, most likely because it was not required at the time of registration of the drug (Phenazolinum, Polfa, Poland) containing ANT mesylate. Species differences in metabolism are often responsible for the difficulties in extrapolating the metabolic data from laboratory animals to humans [19]. Food and Drug Administration (FDA) noted the cases for which clinically relevant metabolites have not been identified during preclinical development [16]. One of the reasons for this is the differences in the CYP P450 enzyme expression [15] among species. Thus, we decided to investigate the metabolism of ANT using human plasma collected from healthy volunteers receiving a standard dose of 100 mg of ANT mesylate, intravenously. We also applied the in vitro model of pooled human hepatocytes. It was very reasonable to perform the metabolic study of ANT as the sensitivity of analytical methods, since the last study reported almost four decades ago [18], has been improved enormously. In this study, liquid chromatography coupled with high resolution tandem mass spectrometry (LC–HRMS) was used for qualitative and semi-quantitative analysis of ANT metabolites. The study was also aimed to investigate the CYP P450 enzyme isoforms involved in the biotransformation of ANT using cDNA expressed human P450 enzyme preparations co-expressed with human NADPH-CYP P450 reductase (Bactosomes™) and LC-HRMS.

## 2. Results and Discussion

**Figure 1:**
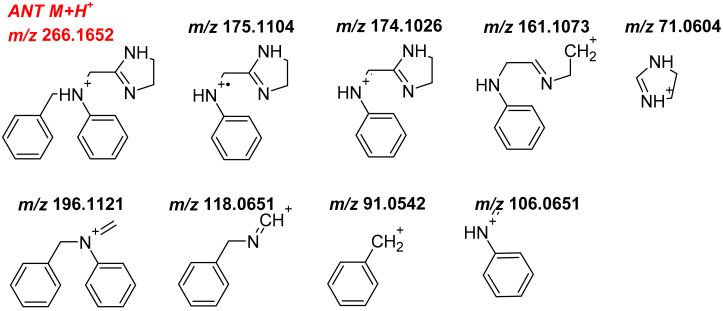
Monoisotopic mass of protonated molecular ion of ANT M+H^+^ (*m*/*z* 266.1652) and its fragment ions obtained with a normalized collision energy of 20, 35, 50 eV using Orbitrap Focus.

Mass spectrometric characterisation of ANT was performed using positive mode ionization and parameters listed in Materials and Methods. Under the conditions applied, ANT formed a protonated molecular ion at *m*/*z* 266.1649 (0.86 ppm, elemental composition C_17_H_19_N_3_ + H^+^). The fragment ions produced in the collision cell were detected at *m*/*z* 196.1124, 175.1107, 174.1025, 161.1075, 118.0654, 106.0653, 91.0544 and 71.0606. Their proposed structures and monoisotopic masses are presented in Figure 1. The tentative structure of metabolites was proposed by comparing *m*/*z* values of the fragment ions of each metabolite with those of ANT to define the region in the molecule in which the transformation occurred.

### 2.1. Determination of Metabolites in Plasma of Volunteers and Hepatocytes Cultures

**Figure 2:**
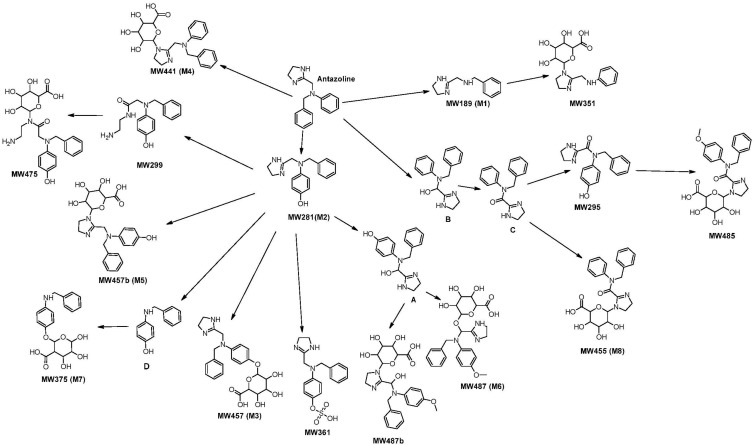
The main pathways of ANT metabolism and putative structure of Phase I and Phase II metabolites. The main metabolites are marked as **M1**–**M8**, other metabolites are labelled with molecular weights (MW). Possible intermediate products were marked A–D. **A**—monoisotopic mass: 297.1477 Da, detected in the plasma of volunteers but did not fulfill the criteria of metabolite selection (see Section 2.2); **B**—monoisotopic mass: 281.1528 Da, not detected in the plasma of volunteers, but detected in CYP in vitro metabolism study (CYP2C19), **C**—monoisotopic mass: 279.1371 Da, detected in the plasma of volunteers but did not fulfill the criteria of metabolite selection (see Section 2.2), **D**—monoisotopic mass: 199.09971 Da, not detected in this study, but described by Ali et al. (1981).

The analysis of chromatograms of extracted plasma from volunteers made it possible to identify 15 potential metabolites of ANT. Their MS data are presented in Table 1 and their tentative structures in Figure 2.

**Figure A1:**
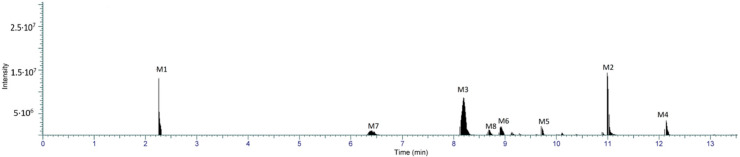
The example chromatogram of metabolites extracted from plasma of volunteers after administration of antazoline. Only the most prominent metabolites were shown.

Other parameters like the ring and double bond (RDB) values, hydrogen vs. carbon atoms ratio (H/C) values, spectral similarity scores between theoretical and measured isotopic patterns (SFit) and matched intensity percentage of the theoretical pattern (Pattern Cov.) are presented in Table A1. The typical chromatogram of the most prevalent metabolites is shown in Figure A1. Most of the metabolites found in the plasma of volunteers were detected as well in an in vitro assay with human hepatocytes (the appropriate annotation is shown in Table 1). Some of the detected compounds are Phase I metabolites including **M1**, **M2**, and metabolite of MW295 as well as MW299. Some of the detected products are Phase I metabolites, additionally methylated and glucuronidated, including **M6**, MW485, and MW487b. Some of them are conjugated with glucuronic acid (ANT or Phase I metabolite) (**M3**, **M4**, **M5**, **M7**, **M8**, MW351, MW475) or with sulfuric acid (MW361) only. The main pathways of ANT metabolism are shown in Figure 2. **M1** is the metabolite of ANT that results from the cleavage of the C-N bond and removal of phenyl. In Phase II metabolism, **M1** forms *N*-glucuronide MW351. In an alternative path, ANT directly undergoes Phase II metabolism, producing *N*-glucuronide **M4**. The other Phase I transformation of ANT is hydroxylation in the *para* position of phenyl substituent, leading to metabolite **M2**. This metabolite is further conjugated with glucuronic acid to form *N*- or *O*-glucuronide (**M5** and **M3**, respectively) or with sulfuric acid forming sulfate MW361. Alternatively, cleavage of the imidazole ring of metabolite **M2**, followed by hydration gives MW299 and further conjugation with glucuronic acid gives MW475. Since in an in vitro post-reaction mixture hydrated ANT was detected it cannot be excluded that in another pathway ANT is firstly hydrated and then hydroxylated to form MW299. **M2** is also hydroxylated to give metabolite A and then glucuronidated followed by methylation to form **M6** and MW487b. Compound A was detected in the plasma of volunteers but it did not fulfill the criteria of metabolite selection as a lower increase in peak area than expected for a metabolite was recorded. ANT is also hydroxylated at the alpha position to the imidazole ring, producing compound B. This is then hydroxylated to the rvespective ketone C. Compound B was not detected in an in vivo study, but it was found in the post-reaction mixture with CYP2C19. Compound C was detected in the plasma of volunteers but did not fulfill the criteria of metabolite selection. Compound C was then conjugated with glucuronic acid to give **M8** or hydroxylated to form MW295 followed by methylation of phenolic hydroxyl and glucuronidated to give MW485. However, MW295 may be formed by oxidation of compound A as well. Another metabolite of ANT might be a compound D, as **M2** without 2-methyl-4,5-dihydro-1*H*-imidazole fragment. Glucuronidation of compound D resulted in the formation of metabolite **M7**. Compound D was not detected in this study, but it was reported previously as a rabbit metabolite of ANT [18].

**Figure 3:**
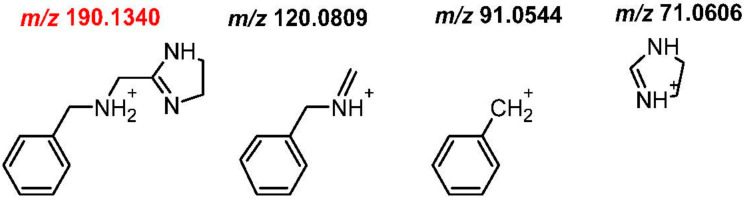

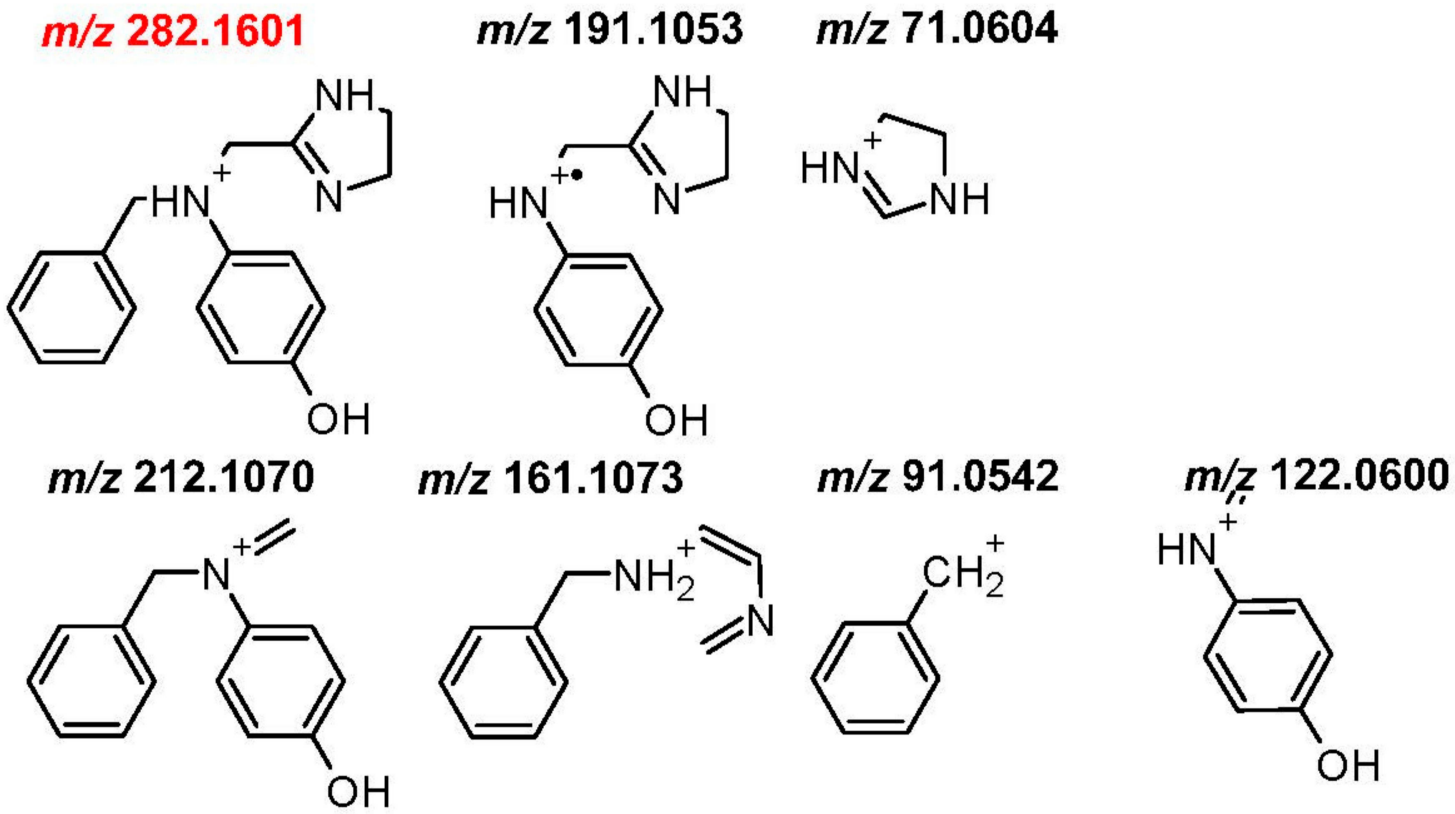
Monoisotopic mass of protonated molecular ion (M+H^+^) of the main ANT Phase I metabolites: **M1** (*m*/*z* 190.1340) and **M2** (*m*/*z* 282.1601) and their fragment ions obtained with a normalized collision energy of 20, 35, 50 eV using Orbitrap Focus. Pseudo-molecular ions of M1 and M2 were marked in red.

The structure of most of the metabolites was confirmed by their fragmentation patterns. The fragment ions of protonated **M1**, produced in the collision cell, were detected at *m*/*z* 120.0811, 91.0545, 71.0607. Their monoisotopic masses and structures are shown at Figure 3.

**Figure 4:**
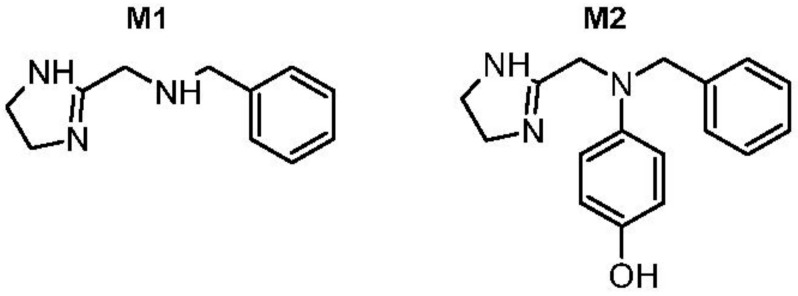
The structures of the two most abundant Phase I metabolites of ANT: **M1** and **M2**. The structure of **M2** was confirmed by chemical synthesis.

The postulated fragmentation pathway of the second most important Phase I metabolite (**M2**) was similar to that of ANT, resulting in ions *m*/*z* 212.1075, 191.1055, 122.0603 by the addition of 16 Da at *m*/*z* 196, 175, 106. Other detected fragments were the same as for ANT, at *m*/*z* 161.1074, 91.0545, and 71.0607 (Figure 3). This indicated that the oxidation occurred at phenyls or methyl that were neighboring imidazole (Figure 4). The comparison of the retention time and fragmentation pattern of **M2** and that of synthesized hydroxyl ANT derivative (to be published elsewhere) confirmed the oxidation at the *para* position in phenyl (Figure A3).

**Figure A2:**
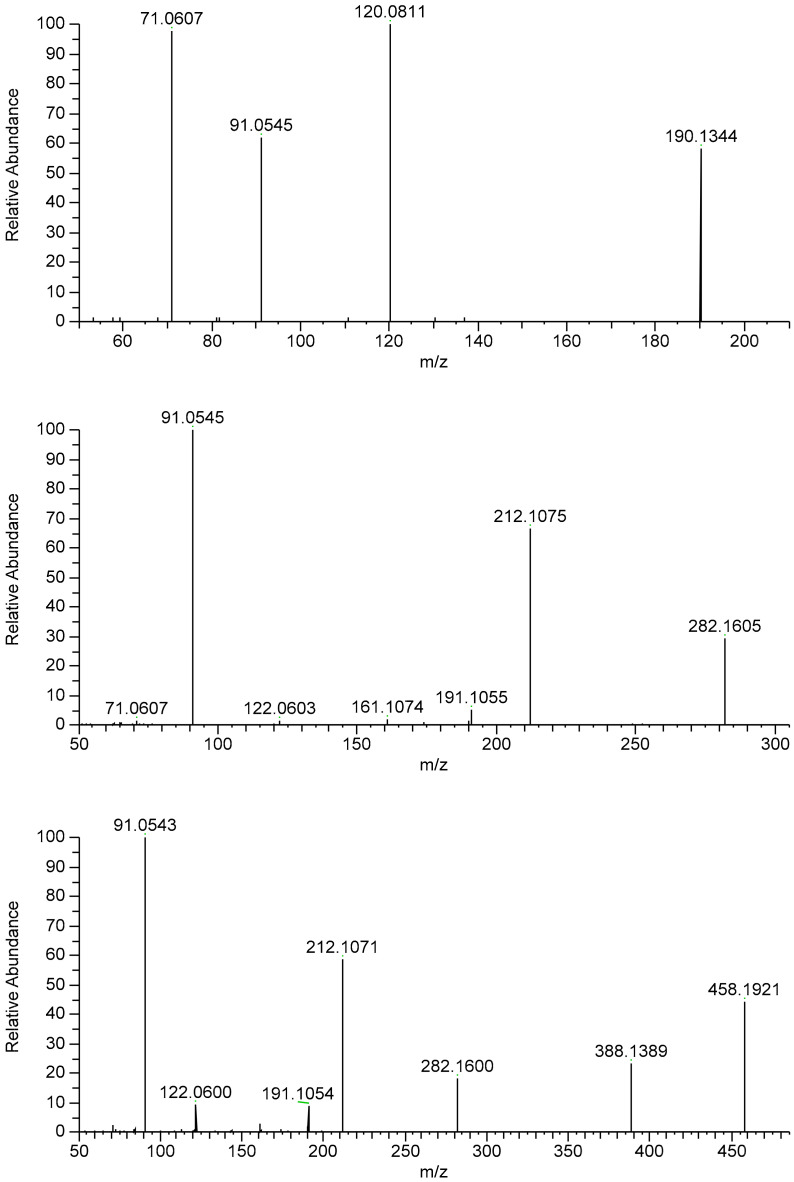

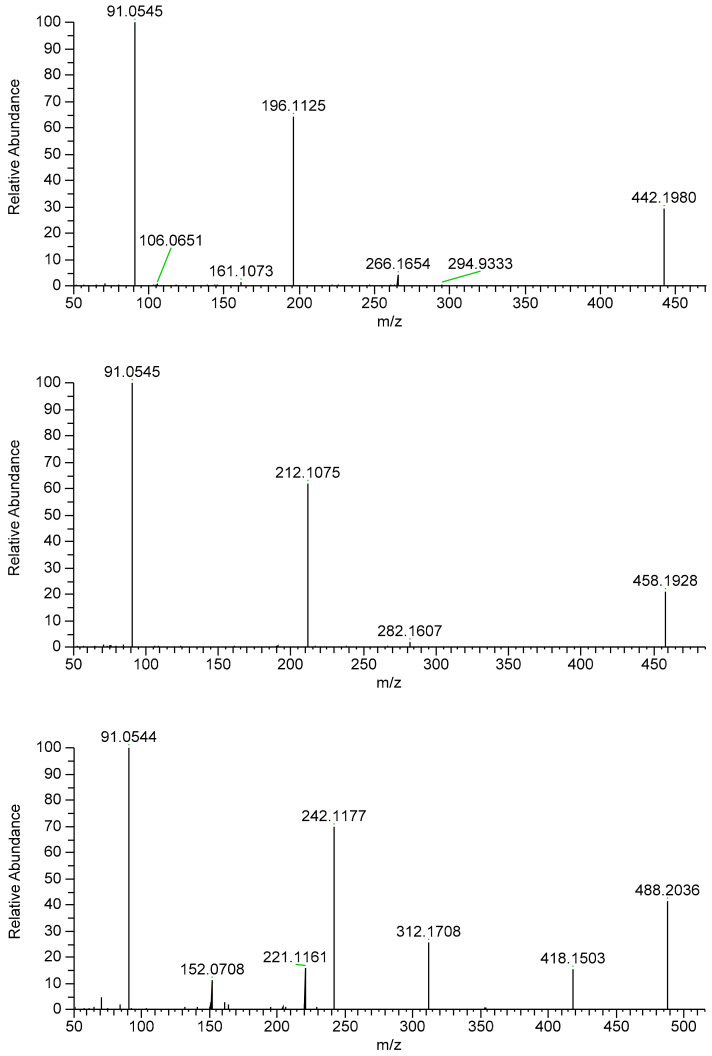

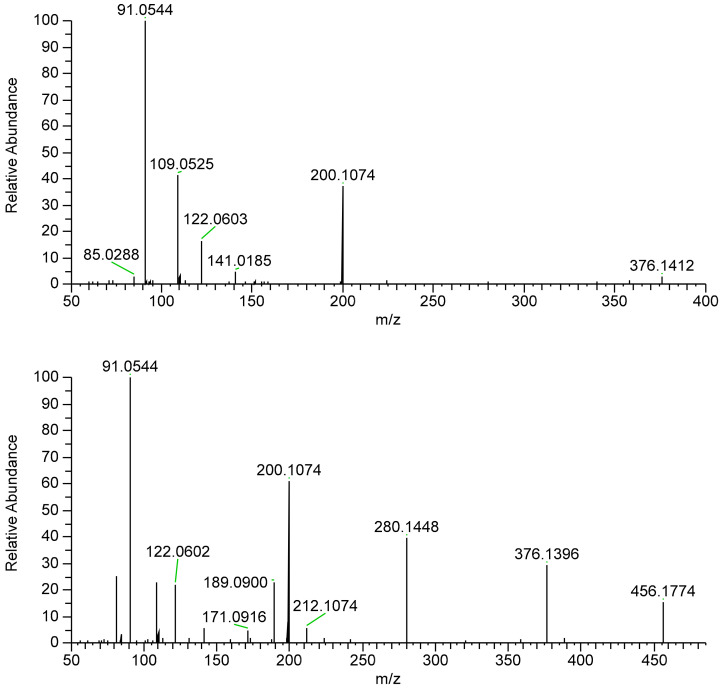
The MS2 spectra of the most prominent antazoline metabolites.

**Figure A3:**
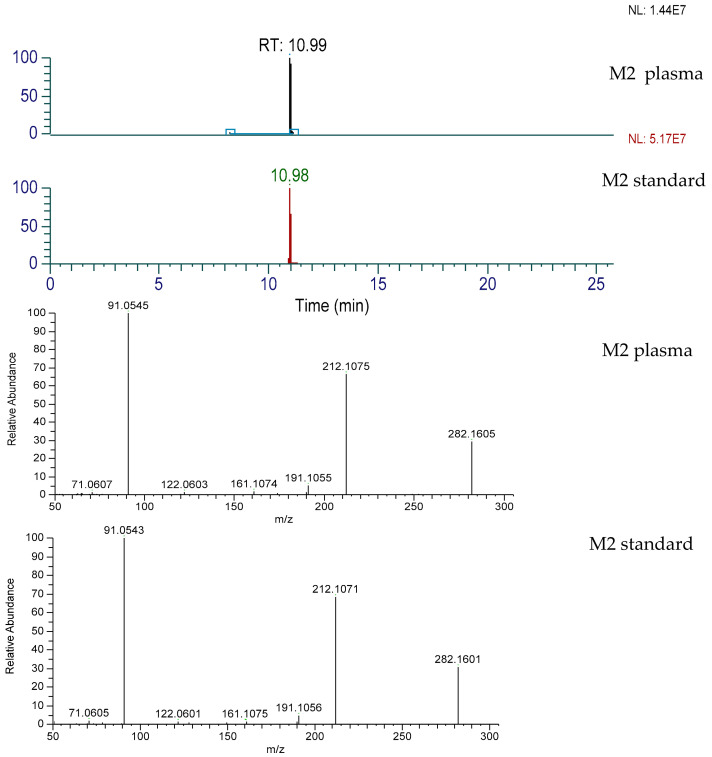
Chromatograms and MS2 spectra of MW281 (M2): synthesized and detected in plasma of volunteers after administration of antazoline mesylate.

One of the most prevalent Phase II metabolite of ANT was *N*-glucuronide, **M4**. The MS2 ions were identical with that of ANT at *m*/*z* at 266.1654, 196.1125, 118.0656, and 91.0544, 71.0606 (Figure A2). The second Phase II metabolite of great importance was *O*-glucuronide of hydroxy ANT (**M3**). We observed the fragments similar to those in the hydroxy ANT (**M2**) spectrum at *m*/*z* 282.1600, 212.1071, 122.0600, 91.0543, 71.0606. Additionally, ion at *m*/*z* 388.1389 formed by the addition of 176 Da to *m*/*z* 212, observed in the spectrum of **M2** was detected. The other fragmentation pathway was observed for *N*-glucuronide of hydroxy ANT (**M5**). None of the fragments contained the glucuronic acid moiety. The main fragments were *m*/*z* 282.1607, 212.1075, 122.0600, 91.0545 and 71.0606 (Figure A2).

**Figure 5:**
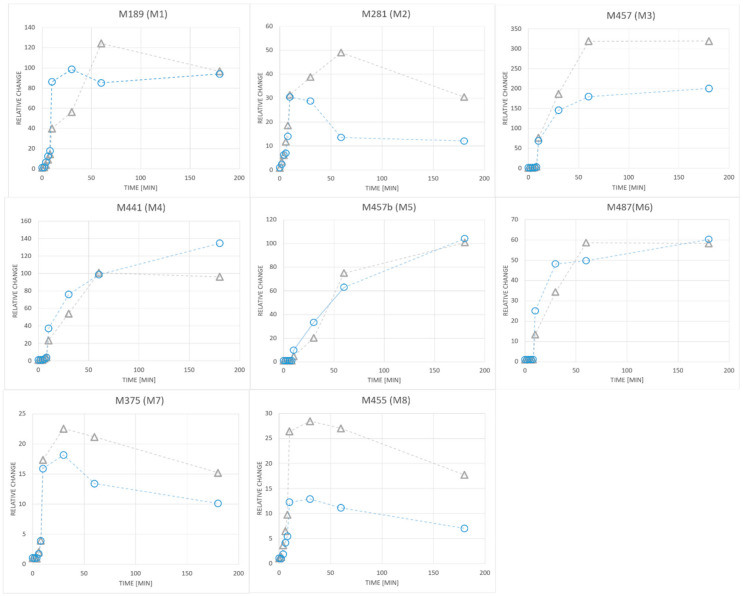
The relative changes of peak areas of metabolites in plasma of two volunteers after administration of ANT. The molecular weights of metabolites are given in Table 1. Only the compounds with increased intensities greater than 10, in at least three sampling points, are presented.

The kinetics of changes in the intensity of ions of selected metabolites (a relative increase in peak area greater than 10 in at least three sampling points) are presented at Figure 5.

**Figure 6:**
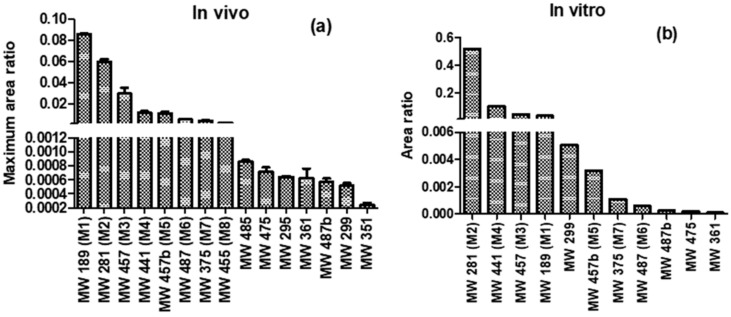
(**a**) The relative maximum peak area (maximum metabolite area divided by the maximum peak area of ANT) of detected metabolites in plasma after administration of ANT (**b**). The relative peak area (the metabolite area divided by the peak area of ANT) of metabolites detected after 120 min of incubation with pooled human hepatocytes.

The maximum peak areas of the metabolites detected (relative to ANT peak) in plasma and hepatocytes culture were presented in Figure 6. In the plasma, the highest peak area was recorded for **M1** (ANT without phenyl) followed by **M2** (hydroxy ANT), **M3** (glucuronide of hydroxyl ANT), and **M4** (*N*-glucuronide of ANT), whereas for in vitro assay the most prominent metabolite was **M2**, followed by **M4**, **M3**, and **M1**.

The metabolite of ANT described by Ali et al. [18], *p*-benzylaminophenolate, with the monoisotopic mass of 199.099, was not detected in our study. However, we detected its glucuronide **M7** (MW375). Another metabolite described previously, *N*-benzylaniline (monoisotopic mass 183.1048), was detected [18], but its concentration was highly correlated with the ANT peak area (r = 0.92, *p* < 0.05). Thus, it was not considered in this paper. The differences in the pattern of metabolites observed in our study might result from the different metabolisms among various species.

### 2.2. In Vitro Analysis Using Various CYP Isoforms

**Figure A4:**
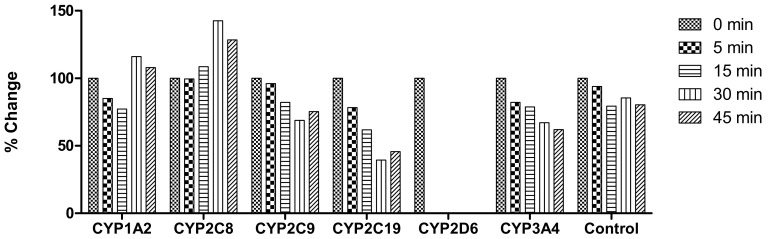
Changes of ANT peak area after ANT incubation with cytochromes: CYP1A2, CYP3A6, CYP2C8, CYP2C9, CYP2C19 and CYP2D6 expression systems (Bactosomes™). In the control sample, no CYP P450 enzymes were present.

The metabolism of ANT with CYP2D6 was fast (Figure A4). Less than 1% of the parent compound was detected after 5 min of incubation. The metabolism of ANT with CYP2C19 was much slower. After 5 and 30 min of reaction, 89% and 50%, respectively, of ANT remained unchanged. ANT was found to not to be the substrate for other cytochrome isoforms i.e., CYP1A2, CYP2C8, CYP2C9 and CYP3A6. In the incubation mixture, the two major Phase I metabolites of ANT, i.e., **M1** and **M2**, were detected. **M1** and **M2** were found in CYP2D6 post-reaction mixture. **M2**, together with metabolite B (MW281, Figure 2), compound A (Figure 2), and compound C (Figure 2), was also detected as the product of the reaction catalyzed by CYP2C19. None of the metabolites were observed in the control samples (no CYP P450 enzymes present). CYP2D6 metabolizes more than 25% of clinically used drugs [20]. The CYP2D6 encoding gene is highly polymorphic with more than 100 allelic variants. High polymorphism of the enzyme causes a different rate of drug metabolism. According to the activity of CYP2D6, the population is categorized as poor, extensive, and ultra-rapid metabolizers. In Europe, ultra-rapid metabolizers (i.e., CYP2D6 duplication) are the most frequently detected in the South-East European countries, where represent up to 6% of the population. Substantially lower frequencies are observed in Northern and Central Europe (1.6–0.5%). Globally, CYP2D6 duplication is the most common in North-East Africa and the Middle East with frequencies of 7–16%. Poor metabolizers are more prevalent than ultra-rapid metabolizers and vary between 15.7% in Finland and Turkey, and 33.6% in the Faroe Islands [21]. Thus, the genetic polymorphism strongly influences the ANT metabolism, which should be taken into account in optimization of the therapy for various groups of patients.

## 3. Materials and Methods

### 3.1. Sample Preparation

#### 3.1.1. In Vivo Analysis

ANT mesylate (Phenazolinum, Polfa, Poland) was given intravenously in 100 mg bolus injected during 1 min (the equivalent of 73.4 mg of ANT) to 2 healthy volunteers. Approximately, 2 mL blood samples were collected (sodium citrate as anticoagulant, 3.2%) before and at 2, 4, 6, 8, 10, 30, 60, 180, 240 and 300 min post-dose. Immediately after collection, blood samples were centrifuged at 2000× *g* for 15 min at room temperature and plasma samples were aliquoted and stored at−80 °C before analysis. Plasma samples (0.1 mL) were diluted with the mixture of methanol and ethanol (Merck Millipore, Darmstadt, Germany) (1:1, *v*/*v*, 0.7 mL) containing 0.15 µg/mL of xylometazoline (surrogate standard) (Toronto Research Chemicals, Toronto, Canada) and put at −20 °C for 30 min. Then, samples were centrifuged (10 min, at 2000× *g*), and the supernatant was evaporated to dryness (40 °C). The residue was reconstituted in 10% aqueous acetonitrile (Merck Millipore, Darmstadt, Germany) (0.1 mL) and 10 µL aliquot was injected into the LC-HRMS. The quality control consisted of equal volumes of each sample. Blank solvent and blank control of a matrix sample with and without antazoline was included in the analytical run as well.

#### 3.1.2. In Vitro Analysis

ANT metabolism was studied in vitro by Cyprotex (Alderley Park, UK) according to their internal procedure and was conducted on ANT mesylate in 10-donor mixed gender pooled hepatocytes samples (LiverPool™, BioreclamationIVT) for 120 min. The hepatocytes had documented activity of ethoxycoumarin-O-deethylase (ECOD), uridine 5′-diphospho-glucuronosyltransferase (UGT), sulfurtransferase (ST), CYP1A2, CYP2A5, CYP2B6, CYP2C8, CYP2C9, CYP2C19, CYP2D6, CYP2E1, and CYP3A4. Incubations (500 µL) were performed at a concentration of 3 µM at 37 °C. The cell density was 0.5 × 10^6^ viable cells/mL. The final dimethyl sulfoxide (DMSO) concentration in the incubation was 0.25%. Control incubations were also performed in lysed cells to reveal any non-enzymatic degradation. Samples were removed from the incubation mixture at 0 and 120 min and added to acetonitrile (1:2, *v*/*v*), containing surrogate standard (metoprolol, 0.55 µM), to terminate the reaction. The samples were centrifuged (2500 rpm at 4 °C for 30 min) and the pooled supernatants were analyzed by LC-HRMS.

To determine the possible CYP isoforms involved in the metabolism of ANT, ANT mesylate (1 µM) was incubated with CYP1A2, CYP3A6, CYP2C8, CYP2C9, CYP2C19 and CYP2D6 expression systems (Bactosomes™) by Cyprotex according to their internal procedure. Bactosomes™ (final P450 concentration: CYP1A2 100 pmol/mL, CYP2C8 50 pmol/mL, CYP2C9 25 pmol/mL, CYP2C19 100 pmol/mL, CYP2D6 50 pmol/mL and CYP3A4 25 pmol/mL), 0.1 M phosphate buffer pH 7.4 and ANT were pre-incubated at 37 °C prior to the addition of reduced form of nicotinamide adenine dinucleotide phosphate (NADPH, final concentration 1 mM) to initiate the reaction. Incubations were also performed using control Bactosomes™ (no P450 enzymes present) to reveal any non-enzymatic degradation. Control compounds known to be metabolized specifically by each P450 isoform are included. ANT was incubated for 0, 5, 15, 30, and 45 min with each isoform. The reactions was stopped by transferring an aliquot of the incubate to a quenching solvent, containing surrogate standard (acetonitrile with metoprolol at 0.55 µM), at the appropriate time points, in a 1:3 (*v*/*v*) ratio. The termination plates was centrifuged at 3000 rpm for 20 min at 4 °C to precipitate the protein.

### 3.2. Instrumental Analysis

#### 3.2.1. In Vivo Analysis

Instrumental analysis was performed using a Dionex Ultimate 3000 with a Q-Exactive hybrid quadrupole-Orbitrap mass spectrometer system (Thermo Fisher Scientific, Waltham, MA, USA) equipped with heat electrospray ionization (HESI), an online vacuum degasser, a quaternary pump, an autosampler, and a thermostatic column compartment. The HESI was operated in a positive mode. Full MS scans were acquired over an *m*/*z* 70–600 range with a resolution of 70,000 (*m*/*z* 200). The target value was 3 × 10^6^. Fragmentation was performed in different runs with a normalized collision energy of 20, 35, 50 eV. The tandem mass spectrum was acquired with resolution 17,500 at *m*/*z* 200. The target value was 5 × 10^4^. The ion selection threshold was 8 × 10^3^ counts, and the maximum allowed ion accumulation times were set to auto both for full MS scans and for the tandem mass spectrum. Standard mass spectrometric conditions for all experiments were: spray voltage, 3.5 kV; sheath gas pressure: 60 arb; aux gas pressure: 20 arb; sweep gas pressure: 0 arb; heated capillary temperature: 320 °C; loop count: 3; isolation window: *m*/*z* 1.0; and dynamic exclusion: 6.0 s. For all full scan measurements, lock-mass ions from ambient air (*m*/*z* 445.1200 and 291.2842) were used as internal calibrants. All data collected in profile mode were acquired and processed using Xcalibur 3.0 software (Thermo Fisher Scientific, Waltham, MA, US), and Compound Discoverer 2.1 (Thermo Fisher Scientific, Waltham, MA, US), respectively. Chromatographic separation was achieved with a Kinetex RP-18 column (100 mm × 4.6 mm, 2.6 µm) supplied by Phenomenex (Torrance, CA, USA) equipped with a security guard. The column was maintained at 40 °C at a flow rate of 0.5 mL/min. The mobile phases consisted of HPLC grade water with 0.1% formic acid (Merck Millipore, Darmstadt, Germany) as eluent A and acetonitrile with 0.1% formic acid as eluent B. The gradient (% B) was as follows: 3.5 min–10%; 30 min–90%; 39.5 min–90%. The volume of injection was 10 µL. Every five samples, quality control was run. To validate the analytical precision, a principal component analysis (PCA) was conducted. In the scatter plot of principal components, all quality controls were clustered in two separate groups.

The compound was considered as a metabolite when it met the following requirements:

1. The proposed formula corresponded to products of Phase I or Phase II metabolic biotransformations.
2. The peak area of the compound increased at least five times and at least in three sampling timepoints (*vs.* time 0) with a gradual increase after drug administration in plasma of both volunteers.
3. The difference between experimental and theoretical molecular weight was not higher than 5 ppm.

#### 3.2.2. In Vitro Analysis

Instrumental analysis was performed by Cyprotex using a Waters XevoQTof G2-S system (Milford, MA, USA) equipped with an Acquity Binary Solvent Manager, Acquity Column Manager and 2777 Autosampler (Milford, MA, USA). The electrospray ionization (ESI) source was operated in a positive mode.

Full MS scans were acquired over the *m*/*z* 130–1000 range. Fragmentation was performed with a collision energy ramp from 10 to 40 V. Spray voltage was 0.3 kV and cone voltage 40 V. For all full scan measurements, lock-mass ions of leucine enkephalin (0.4 μg/mL, 10 μL/min) were used as internal calibrants. Chromatographic separation was achieved with a C18 ACQUITY UPLC^®^ HSS T3 column (Thermo Fisher Scientific, Waltham, MA, US) (100 mm × 2.1 mm, 1.8 µm). The column was maintained at 60 °C at a flow rate of 0.4 mL/min. The mobile phases consisted of 10 mM ammonium formate with 0.1% formic acid as eluent A and acetonitrile with 0.1% formic acid as eluent B. The gradient (% B) was as follows: 0.5 min—2%; 10 min—35%; 11 min—95%. The volume of injection was 10 µL. The data was processed using Metabolynx XS (Waters Ltd., Milford, MA, US). The 120-min samples were compared against the 0-min control sample.

To determine the possible CYP isoforms, instrumental analysis was performed using a Dionex Ultimate 3000 with a Q-Exactive hybrid quadrupole-Orbitrap mass spectrometer as described in the in vivo analysis section.

## 4. Conclusions

The present in vitro study revealed CYP2D6 as the main CYP isoform involved in the metabolism of ANT. Since the enzyme is highly polymorphic, the intensities of metabolites in the metabolic profile of ANT in humans can exhibit high inter-individual variation. This should be considered in the selection of the ANT dose in a particular AF treatment. Human metabolism of ANT includes both Phase I and Phase II reactions, which are mainly glucuronidation. In this study, we showed that ANT was metabolized to at least 15 compounds. The most prominent Phase I metabolite of ANT is **M1** (N-benzyl-1-(4,5-dihydro-1H-imidazole-2-yl)methanamine), formed by the removal of phenyl and metabolite **M2** resulting from introducing hydroxyl into phenyl in the *para* position of ANT. **M2**, as the hydroxylated analog of ANT, might be less toxic than ANT due to its higher hydrophilicity, and, as the compound structurally close to ANT, might also reveal similar antiarrhythmic effects. However, since metabolic hydroxylation might lead to compounds that are either less or more potent than the parent drug, further research in this direction is needed. Studies on pharmacological activity, potency, toxicity, and pharmacokinetics of the metabolites would allow us to better understand the activity profile of ANT and to predict its potential clinical application. Ultimately, these studies might result in the development of a new drug substance.

## Figures and Tables

**Table 1:** Experimental molecular weight, the difference between the theoretical and experimental molecular weight, retention time, molecular formula, and the most abundant daughter ions (normalized collision energy of 20, 35, 50 eV) of the tentative metabolites of ANT.

Product	Experimental Molecular Weight [Da]	Retention Time [Min]	Molecular Formula	ΔMass [ppm]	Differences with ANT	Detected in vitro ^1^	MS/MS Ions
M1 (MW189)	189.1263	2.2	C11 H15 N3	−1.58	−6C,4H	yes (3.6)	120.0811, 91.0545, 71.0607
M2 (MW281)	281.1525	11.0	C17 H19 N3 O	−1.10	+O	yes (6.6)	212.1075, 191.1055, 161.1074, 122.0603, 91.0545, 71.0607
MW295	295.1320	10.4	C17 H17 N3 O2	−0.23	−2H,+2O	yes (6.6)	205.0856, 188.0835, 91.0545, 71.0601
MW299	299.1634	11.0	C17 H21 N3 O2	0.06	+2H,2O	yes (6.7)	212.1071, 149.0475, 122.0603, 91.0543, 72.0444
MW351	351.1427	13.5	C16 H21 N3 O6	−0.96	−C,+2H,6O	no	-^2^
MW361	361.1095	11.2	C17 H19 N3 O4 S	−0.19	+S,4O	yes (5.9)	-^2^
M7 (MW375)	375.1318	6.4	C19 H21 N O7	0.13	+2C,2H,7O,−2N	yes (5.6)	358.1306, 224.1069, 200.1074, 122.0603, 109.0525, 91.0544
M4 (MW441)	441.1901	12.1	C23 H27 N3 O6	0.45	+6C,8H,6O	yes (7.1)	266.1654, 196.1125, 161.1073, 91.0545, 71.0606
M8 (MW455)	455.1702	8.7	C23 H25 N3 O7	−1.70	+6C,6H,7O	no	376.1396, 280.1448, 189.0900, 91.0544, 71.0127
M3 (MW457)	457.1849	8.2	C23 H27 N3 O7	0.08	+6C,8H,7O	yes (4.8)	388.1389, 282.1600,212.1071, 122.0600,91.0543,71.0606
M5 (MW457b)	457.1850	9.7	C23 H27 N3 O7	0.32	+6C,8H,7O	yes (5.3)	282.1607, 212.1075, 122.0600, 91.0545, 71.0606
MW475	475.1957	9.1	C23 H29 N3 O8	0.69	+6C,10H,8O	yes (5.1)	458.1936, 300.1702, 282.1605, 212.1072, 122.0602, 91.0544, 71.0607
MW485	485.1795	10.9	C24 H27 N3 O8	−0.45	+7C,8H,8O	no	310.1551, 257.0769, 242.1178, 91.0544, 81.0449
M6 (MW487)	487.1957	8.9	C24 H29 N3 O8	0.49	+7C,10H,8O	yes (5.1)	418.1503, 312.1708, 242.1177, 221.1161, 152.0708, 91.0544, 71.0606
MW487b	487.1957	9.3	C24 H29 N3 O8	0.55	+7C,10H,8O	yes (5.3)	312.1709, 176.1187, 137.0600, 106.0655, 71.0605

^1^ retention time [min] was provided in brackets; ^2^ low quality MS2 spectra.

## Appendix A

**Table A1:** Experimental molecular weights, ring and double bond (RDB) values, hydrogen vs. carbon atoms ratio (H/C) values, spectral similarity scores between theoretical and measured isotopic patterns (SFit), and matched intensity percentage of the theoretical pattern (Pattern Cov.), of the tentative metabolites of antazoline.

Product	Experimental Molecular Weight [Da]	RDBE	H/C	SFit [%]	Pattern Cov. [%]
MW189	189.1263	6	1.4	84	99.88
MW281	281.1525	10	1.1	89	99.91
MW295	295.1320	11	1	92	98.37
MW299	299.1634	9	1.2	98	98.37
MW351	351.1427	8	1.3	82	97.77
MW361	361.1095	10	1.1	76	95.45
MW375	375.1318	10	1.1	96	97.1
MW441	441.1901	12	1.2	65	100
MW455	455.1702	13	1.1	85	99.55
MW457	457.1849	12	1.2	78	100
MW457b	457.1850	12	1.2	85	100
MW475	475.1957	11	1.3	95	95.96
MW485	485.1795	13	1.1	89	95.74
MW487	487.1957	12	1.2	82	99.47
MW487b	487.1957	12	1.2	91	95.74
